# Development and validation of REAGERA-P, a new questionnaire to evaluate health care provider preparedness to identify and manage elder abuse

**DOI:** 10.1186/s12913-021-06469-2

**Published:** 2021-05-19

**Authors:** Johanna Simmons, Marika Wenemark, Mikael Ludvigsson

**Affiliations:** 1 Department of Acute Internal Medicine and Geriatrics in Linköping, and Department of Health, Medicine and Caring Sciences, Linköping University, Linköping, Sweden; 2 Unit of Public Health and Statistics in Region Östergötland, and Department of Health, Medicine and Caring Sciences, Linköping University, Linköping, Sweden; 3 Department of Psychiatry in Linköping, and Department of Biomedical and Clinical Sciences, Linköping University, Linköping, Sweden

**Keywords:** older adults, intimate partner violence, neglect, instrument development, validity, mistreatment

## Abstract

**Background:**

Elder abuse is prevalent and associated with morbidity but often goes unnoticed in health care. Research on the health care response to victims calls for valid measurements. This article describes the development and validation of a questionnaire to evaluate health care provider preparedness to care for older adults subjected to abuse, the REAGERA-P (Responding to Elder Abuse in GERiAtric Care – Provider questionnaire).

**Method:**

REAGERA-P was developed in phase I. The questionnaire includes a case vignette, self-efficacy scales for identifying and managing elder abuse cases and cause for concern as well as organizational barriers when talking with older patients about abuse. Content validity was ensured by a review committee, and cognitive interviews were conducted to ensure face validity and to examine cognitive processes to ensure comprehension. REAGERA-P was then administered to health care providers (n = 154, response rate 99 %) to test for construct validity. Factor analysis was performed, and internal consistency was tested for the self-efficacy scales. Convergent validity was tested by investigating associations between relevant variables. Some items were revised in phase II, and new cognitive interviews were performed. Parts of the questionnaire were tested for responsiveness by administering it to medical interns (n = 31, response rate 80 %) before and after an educational intervention.

**Results:**

REAGERA-P showed good content and face validity. The factor analysis revealed two factors: one for asking questions about abuse (Cronbach’s α = 0.75) and one for managing the response to the questions (Cronbach’s α = 0.87). Results suggest good convergent validity for the self-efficacy scales and for questions about cause for concern and organizational barriers. The responsiveness of the self-efficacy scales was good: the mean on the scale for asking questions (range 0–30) was 15.0 before the intervention and 21.5 afterwards, the mean on the scale for managing the response (range 0–50) was 22.4 before the intervention and 32.5 afterwards.

**Conclusion:**

REAGERA-P is a new questionnaire that can be used to evaluate health care provider preparedness to identify and manage cases of elder abuse, including educational interventions conducted among staff to improve health care responses to victims of elder abuse. This initial testing of the questionnaire indicates that the REAGERA-P has good validity.

**Supplementary Information:**

The online version contains supplementary material available at 10.1186/s12913-021-06469-2.

## Background

A global meta-analysis has found that 15.7 % of community-dwelling older adults have been subjected to some form of elder abuse in the past 12 months [1]. Dependence on other people for one’s physical or mental wellbeing is a strong risk factor for elder abuse [2, 3]. Therefore, it is not surprising that the rates of the prevalence of abuse have been reported to be much higher among older adults suffering from dementia or living in an institution, around 50 % in some studies [4–6]. Elder abuse has repeatedly been associated with different forms of morbidity and increased hospitalization and admission to skilled nursing facilities [3, 7–9].

For some older adults, the health care system might be the only place to establish contact with professionals outside the circle of abuse, and therefore, the health care system has the potential to act as an important source of support for victims [10, 11]. However, many victims of elder abuse are reluctant to share their stories of abuse with health care providers, and health care providers have been reported to be reluctant to ask patients questions about abuse or to report cases to authorities. Hence, many victims go unnoticed by health care providers [12–19].

Barriers to identifying and managing elder abuse in health care have been found on the personal level, on the organizational level, and on the system level [11, 14, 15, 20]. Examples of barriers on the personal level are when health care professionals are unaware of elder abuse or unsure of what constitutes abuse, when they are concerned that asking about abuse would have a negative impact on their relationship with the patient, or when they lack confidence in their ability to manage abuse cases [11–16, 20–23]. Some reported that barriers on the organizational or system levels include a lack of time to deal with such a complex issue or lack of clear referral pathways to effective support systems [11, 14, 20, 23–25].

More research into the area is needed to improve the identification and management of elder abuse in health care, and there is a need to create effective educational interventions for professionals. Studies of health care professionals’ preparedness to care for victims of elder abuse and studies of educational interventions call for valid measurement tools, which are lacking in Sweden and internationally. When health care professionals’ attitudes and knowledge about elder abuse are studied, results are often reported without the measurements that were based on validated questionnaires [15]. Similarly, many training programs do not include validated instruments to measure the effect of the program [26, 27]. Evaluations of interventions are also often limited to knowledge about elder abuse and satisfaction with training on the topic. Changes in attitudes and practices are typically not considered [26].

Two instruments using case vignettes have, however, been validated and show good internal consistency. Both questionnaires focus on recognizing elder abuse and knowledge about how to act when cases of elder abuse are suspected or identified, but they do not include perceived competence in doing so [16, 28]. Different version of questionnaires inspired by Clark-Daniels et al. [29] and Kennedy [14] have been widely used, but the validity and reliability of those questionnaires have either not been reported at all or only very briefly [15, 24, 29–34]. Those questionnaires have generally included questions about experience of managing elder abuse, knowledge about and attitudes towards elder abuse, and knowledge about mandatory reporting. The latter is not relevant in settings where there is no mandatory reporting legislation, including Sweden. Questions about skills and confidence in identifying and managing abuse cases are only briefly included in one version [31].

In summary, there is a need for a validated questionnaire that measures preparedness, that covers both practices and perceived skills among health care professionals, and that considers known barriers to identifying and managing elder abuse [11, 14, 15, 20]. The aim of this study was, therefore, to develop and test a questionnaire that can be used to measure the preparedness to identify and manage elder abuse. Such a questionnaire would be useful for studies evaluating health care provider preparedness to care for victims and would also be a key component for measuring the effect of educational interventions, which indirectly could improve the identification and management of elder abuse.

## Study overview

**Fig. 1:**
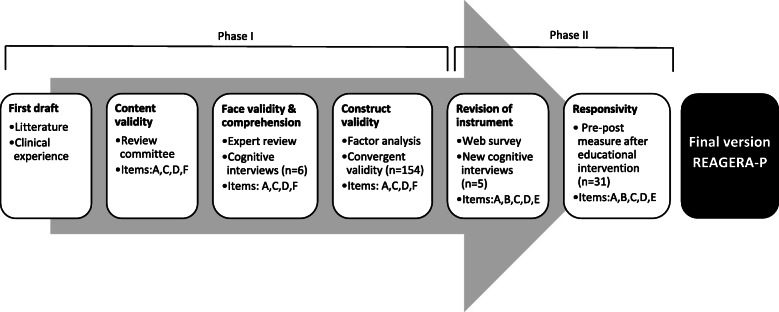
The process of developing and testing REAGERA-P. The letters given represents the items from the final version of REAGERA-P that was included in each step. Items can be found in the supplementary file. A = Background characteristics; B = Case vignette (new); C = Cause for concern D = Self-efficacy scale; E = Own previous experiences; F = Organizational barriers and potential improvements

The Responding to Elder Abuse in GERiAtric care (REAGERA) project was started at the department of acute internal medicine and geriatrics at a Swedish university hospital in 2017. A screening instrument to identify life-time experiences of abuse and elder abuse among older patients was developed and tested in the first part of the project, involving patients (REAGERA-S) [35]. The present study concerns another part of the project; to develop and test a questionnaire to evaluate provider preparedness to identify and manage elder abuse (the REAGERA -P, where P stands for provider). This was conducted in two phases: Phase I: Develop and test the validity of the questionnaire conducted in 2017–2018; and Phase II: Revise the questionnaire and test of responsiveness conducted in 2020. As the second phase was heavily dependent on the results from the first phase, we will present the methods and results of phase I first, followed by methods and results of phase II. An overview of the study procedure and populations and the steps taken to ensure the validity of the questionnaire is presented in Fig. 1.

## Phase 1: Development and validity test

### Method phase I

#### Development of REAGERA-P

The first draft of the REAGERA-P was developed by the first author. It was inspired by two previous instruments in Swedish that are used to measure health care provider preparedness to care for victims of intimate partner violence of all ages [36–38]. Questions were, however, revised to better reflect clinical encounters with older adults. Changes were made primarily based on a literature review and were inspired by the clinical experience of the authors and by comments from colleagues. The following previous literature was used when designing the first draft of the questionnaire.

Awareness and knowledge about elder abuse have been reported to be low among health care providers both in Sweden and internationally [12–14, 31]. This may lead to symptoms being overlooked by professionals, e.g., bruises on an older adult using oral anticoagulants may be glossed over instead of being interpreted as a possible sign of abuse. Case vignettes have previously been successfully used to assess health care provider’s ability to identify elder abuse cases [16, 28].

Self-efficacy was included as the primary measure of respondents’ own assessment of their capability to identify and manage elder abuse. Self-efficacy pertains to people’s perceived capability to perform a certain task and is a concept separate from general self-confidence [39]. Theoretically, people who have a strong self-efficacy for a certain task will be more likely to perform that task successfully. When faced with difficulties people with high self-efficacy are more likely to persevere in their efforts, while people with low self-efficacy will give up more easily [39].

One cause for concern often raised by health care professionals is that enquiring about elder abuse would have a negative impact on their relationship with the patient, e.g., fear of upsetting or alienating the patient if a situation or action is labeled as abuse [11, 15, 20]. Concern that asking about abuse would lead to negative consequences for the patient, e.g., retaliation from the perpetrator, has also been repeatedly reported [15, 20, 21].

Barriers on the organizational or system level include time constraints, uncertainty about responsibilities, and a lack of clear referral pathways between services [11, 20, 31]. Health care professionals are also often uncertain about how to report cases and do not always feel confident that the support systems will be sufficient for older adults subjected to abuse [11, 14, 20, 31].

#### Content validity

To ensure content validity, a review committee met to discuss the content of the first draft. The committee consisted of eight members: three physicians, two occupational therapists, one neuropsychologist, and two nurses. Six of the members combined clinical work in a geriatric department with research about geriatrics or violence and abuse. The two nurses were full time academics with experience of researching elder abuse, intimate partner violence and health care interventions. The committee was asked to review the questions of the first draft for content and relevance beforehand and at the meeting the draft was debated. The group agreed that the content was relevant and captured the primary areas of interest, and no major changes were made.

#### Face validity and comprehensibility

An expert review was conducted to ensure face validity. It was carried out by the second author, who is a specialist in designing questionnaires and the respondent’s perspective in survey research. The first and second authors adjusted the questionnaire to improve the wording and structure of the questions. This resulted in a new structure of the questionnaire consisting of seven parts: (A) background characteristics, 9 items; (B) case vignettes with follow-up questions intended to measure awareness and willingness to ask questions, 8 items; (C) self-efficacy scale, 8 items; (D) cause for concern, 5 items; (E) own previous experience, 3 items; (F) organizational barriers and professional responsibility, 6 items; and (G) potential improvements at the workplace, 2 items.

Six cognitive interviews were conducted to examine cognitive processes and thereby ensure comprehensibility of REAGERA-P. Participants in the interviews were health care professionals with different experiences of managing patients who were victims of elder abuse as well as different professions and workplaces (one nurse and one psychologist at a psychiatric clinic, one resident physician in general medicine, and three nurses in geriatric care). The interviews were conducted to ensure that the questions were well understood and that respondents perceived the questions as relevant and easy to answer. The interviews began with the think-aloud technique, meaning that the informants were asked to respond to the questionnaire and verbally express their thinking while doing so. Thereafter probes, e.g., “can you elaborate more about…,” were used to further understand the informants’ cognitive processes while answering the questions. The first author conducted five interviews, and the second author conducted one. The third author participated as an observer in two of the interviews. Careful notes were taken by the interviewing author (n = 4) and, when applicable, by the observing author (n = 2).

The questions were generally well understood by informants, but some ambiguities in interpretation emerged during the interviews, which led to rephrasing and clarifying some questions. For example, item no. 2 on the self-efficacy scale pertains to the respondent’s ability to ask an older patient who does *not* show any signs of abuse about abusive experiences. The item is intended to be about feeling capable of asking questions, i.e., how to frame questions when there are no clear symptoms or signs to proceed from. However, some informants understood this item as “would it *occur to you* to ask an older patient about abuse if they do not show any signs of abuse”; The wording was, therefore, modified, and the last three informants understood the question in the intended way. Another example of revision was the question about the overall experience of the respondent of talking to older patients about abuse. This question was initially found to be unclear to the informants and, therefore, a parenthesis was added to clarify the intent of the question. The final version reads: On a general level, how were your perceptions of the meeting with older patients who told about abuse? *(In terms of your perceptions of the conversation, how satisfied were you with the handling of cases, etc.)* Response options were (a) mainly positive, (b) mainly negative, and (c) No experience.

After the cognitive interviews, no further test of validity was performed for the questions about background characteristics, own previous experience, professional responsibility, or potential improvement. For this reason, these questions will not be discussed further. The items for which additional validity testing was performed are presented in Table 1.

**Table 1:** Items in REAGERA-P

**Case vignettes – Awareness of and attitudes to asking**
Four cases with different main complaints: (a) bruises, (b) overdose antidepressants, (c) frequent attender with unclear symptoms, (d) chronic disease suddenly much worse. Two follow up questions
- How likely is it that you will ask [patient name] about abuse at any point during the conversation? *Response options: Not at all likely, not very likely, fairly likely, very likely*
- Do you think that any of the care staff should ask [patient name] questions about abuse? *Response options: Yes, only based on the case, Yes, if any further signs, Yes, if he/she implies that he/she is a victim, No, it is best for him/her to take the initiative.*
**Self-efficacy scale**:
At present, how would you manage to do the following things in your work?
- Eight specific tasks chosen to represent challenges concerning identifying and managing cases of elder abuse. Items are presented in Table 4 (factor analysis). *Response options: Scale 0–10 with 0 denoted as “*Would manage it very poorly*” and 10 as “*Would manage it very well.*”*
**Cause for concern**
How worried are you about the following things when it comes to asking older patients questions about abuse?
- That the patient reacts negatively if I ask questions - That the patient-care provider relationship will be negatively impacted if I ask questions - That the patient’s situation will get worse if I ask questions - That I myself will end up in a dangerous or threatening situation if I ask questions - That I will not be able to offer the patient a good follow up *Response options (same to all questions): Not at all concerned, a little concerned, somewhat concerned, very concerned.*
**Organizational barriers**
20. In your current work situation, how often do you have time to bring up the issue of abuse of older people with your patients if you would like to? *Response options: Never, rarely, often, always* 21. If you would like help to handle the situation when an older patient tells you about abuse, do you know who at your workplace you could turn to? *Response options: Yes, No*
22. How do you think the preparedness (a) at your workplace is (b) in society is for taking care of older patients subjected to abuse? *Response options: Very good, Fairly good, Somewhat inadequate, Very inadequate, Don’t know what preparedness there is*
23. Do you know what you should do to document what patients tell you about abuse in a correct and secure way in the medical record? *Response options: Absolutely, To a great extent, To some extent, Definitely not*
24. Do you think you have enough legal knowledge, for example about when and to whom one can/must report if an older patient is mistreated and what secrecy rules apply? *Response options: Absolutely, To a large extent, To some extent, Not really*

#### Test of construct validity

##### Study population

We performed a study among health care providers to test the construct validity of the questions. Eligible for the study were all staff (n = 166) participating in a continuing education program at one acute internal medicine and geriatric clinic at a university hospital in Sweden in 2018. During one session participants were given the opportunity to respond to the REAGERA-P followed by a brief introduction to the REAGERA project. The response rate was 99 % (n = 165), but eleven respondents reported that they did not have any direct contact with patients at work, and they were therefore excluded. This was the only exclusion criteria which left a total sample of 154. No incentive for participation was given and respondents completed the questionnaire in about 10–15 min. Background characteristics of the sample can be found in Table 2.

**Table 2:** Background characteristics of respondents in phase I (*n* = 154) and phase II (*n* = 31)

		Phase I		Phase II	
		n	%	n	%
Sex	Female	139	90.3	22	71.0
	Male	15	9.7	9	29.0
Age	< 34 years	65	42.2	25	80.6
	35–49 years	34	22.1	6	19.4
	> 50 years	55	35.7	-	-
Profession	Assistant nurse	64	41.6	-	-
	Nurse	58	37.7	-	-
	Physician	21	13.6	31	100
	Other	11	7.1	-	-
Previous training about abuse	No, do not remember	73	47.4	4	12.9
	Elder Abuse only	20	13.0	0	
	Violence in close relationships only	40	26.0	20	64.5
	Both elder abuse and violence in close relationships	21	13.6	7	22.6
Ever talked to older adults about abuse	No, do not remember	77	50.3		
	Yes	76	49.7		
Talked to older adults about abuse last 6 months	No, do not remember			20	64.5
	Yes			11	35.5

Note: Missing cases 0–1

##### Statistical analysis

First, descriptive statistics were used to investigate if the items produced a variety of different responses. Bandura [39] recommends this for self-efficacy scales as a way to ensure that the task considered is complicated enough. A diversity of answers is also desirable for other measurements to avoid ceiling and floor effects. Thereafter we used explanatory factor analysis to test the homogeneity of and correlation between items intended to be used as scales (self-efficacy and case vignettes). We used a principal axis factoring analysis and a direct oblimin rotation because we expected extracted factors to be correlated. The questions about concerns and organizational barriers were intended to measure several different aspects and not one underlying concept. Consequently, we did not perform factor analysis or tests for internal consistency for those items.

There was no available previous instrument that could be used to assess the convergent and discriminant validity of REAGERA-P. Instead, we used the alternative option of testing for logical associations between items within REAGERA-P to investigate if the items performed in predictable ways. We expected the following associations:

- Experience is theorized to affect the level of self-efficacy, which is also likely to correlate with cause for concern. We, therefore, expected that respondents reporting positive experience of talking to patients about elder abuse would have: (A) higher self-efficacy, and (B) less concern than those reporting negative or no experience at all. We also expected that (C) self-efficacy would be negatively correlated with concern, i.e., respondents with strong self-efficacy would report less concern.
- Respondents with experience of talking to older patients about abuse (whether positive or negative experience) were expected to be more aware of the preparedness (A) of society and (B) of the clinic to manage cases.
- Feeling confident about expectations and obligations when handling cases of elder abuse, and feeling confident about knowing where to get help if needed are also likely to influence self-efficacy. We, therefore, expected a positive correlation between self-efficacy and: (A) perceived capability of documenting in a correct way, (B) perceived knowledge about legislation, and (C) perceived collegial support, i.e., knowing which colleague to turn to if help was needed to manage cases. Asking questions about abuse is reported to be considered as time consuming. We, therefore, expected that providers who reported to have sufficient time to ask questions to also be more confident that they could ask questions in a good way, i.e., have a higher self-efficacy in asking questions.

The following tests were applied to assess associations: (A) a T-test for independent samples was used to compare the means on scales between groups of respondents, (B) the Spearman correlation was used for ordinal variables, and B) the Chi-square test for linear trend was used to test for associations between nominal and ordinal variables. Pearson’s chi-square test was used for 2 × 2 nominal crosstabs. The significance level was set at *p* = 0.05 and we used bootstrapping with 95 % Bias corrected accelerated confidence intervals (BCa CI) for 1000 samples.

### Results phase I

#### Test of construct validity

##### Distribution of answers

The distribution of answers to the case vignettes was rather limited. Only a few respondents reported that it was rather or very likely that they would ask questions. Around 60–70 % of respondents answered that the patient should be asked questions only if there were more signs of abuse than presented in the vignette or if the patient themselves implied that they had been abused.

All items on the self-efficacy scale showed a diversity in answers. Item four, about supporting an older adult during a conversation, ranged from 1 to 9, and all other items ranged from 0 to 10. The mean values for each item ranged from 2.93 (SD 2.24) for item 2 to 7.09 (SD 1.98) for item 4.

Few respondents reported to be very concerned about a negative reaction from the patient (*n* = 5), that the patient-care provider relationship would be negatively affected (*n* = 3), that the patient’s situation would worsen (*n* = 3), or that they themselves would end up in a dangerous situation (*n* = 4). “Very concerned” was, therefore, merged with “Somewhat concerned” for these items.

A large percentage of respondents did not know about the preparedness of the clinic (42 %) or of society (41 %) to handle cases of elder abuse. Very few respondents thought that the preparedness of the clinic (3.2 %) or society (1.3 %) was very good. Only 2.6 % of respondents felt “absolutely” confident that they were familiar with current legislation, and therefore, that category was merged with the category “to a large extent” confident about legislation.

##### Factor analysis - Case vignettes

We expected to find one or two underlying constructs for the questions used in the case vignettes that would capture awareness of elder abuse and attitudes towards asking questions. However, the factor analysis revealed three factors and an unclear factor loading pattern. The Cronbach’s alpha was poor for the resulting factors (0.41–0.61).

##### Factor analysis - Self-efficacy scale

Two factors were retained in the factor analysis of self-efficacy as they had eigenvalues over Kaiser’s criterion of 1 (factor 1 = 3.93, factor 2 = 1.43). Together they explained 67 % of the variance, and the scree plot verified the choice of retaining two factors as it had a clear point of inflection at 3. The first factor below the threshold had an eigenvalue of 0.62, which further supported our choice. Although we had a rather small sample size, the sampling adequacy for the factor analysis was acceptable as verified with the Kaiser-Meyer-Olkin measure (KMO = 0.82).

Table 3 shows factor loading (pattern matrix) after rotation and findings suggest that one factor concerns self-efficacy in asking question about abuse, and one factor concerns self-efficacy in managing the response. As expected, the two factors were correlated (0.48), which is also theoretically motivated since it is likely that self-efficacy in asking questions and managing the response are associated. Because of the rather strong correlation between these factors, we concluded that the self-efficacy questions could be handled as one main scale (range 0–80) that can also be divided into two subscales; (1) self-efficacy in asking questions (item 1–3, range 0–30) and (2) self-efficacy in managing the response (items 4–8, range 0–50). The overall internal consistency reliability of the self-efficacy scale was good as assessed with Cronbach’s alfa (all items = 0.85, subscale for asking questions = 0.75, subscale for managing response = 0.87).

**Table 3:** Result of the exploratory factor analysis of the self-efficacy scale in REAGERA-P. Overall question: At present, how would you manage to do the following things in your work?

		Rotated factor loadings	
Item no.		Asking questions	Managing the response
1	Asking questions about abuse to an older patient who has clear indications of now being, or having previously been, subjected to abuse.	.**75**	
2	Asking questions about abuse to an older patient who has no clear indications of now being, or having previously been, subjected to abuse.	.**66**	
3	Ensuring you are able to ask questions about abuse in private to an older patient who has a relative who insists on being present during all contact.	.**73**	
4	In conversation, providing support to an older patient who tells about abuse.	0.17	.**58**
5	Helping an older patient subjected to abuse on to the right body in healthcare, or to the right support function in society		.**85**
6	Helping an older patient subjected to abuse to make a report to the police or social services.		.**81**
7	Helping and supporting an older patient subjected to abuse, who does not currently want to change his or her situation.		.**82**
8	Handling the meeting with an older patient who says no to questions about abuse, but where you still have strong suspicions that the patient is subjected to abuse.	0.19	.**64**
	Eigenvalues	1.43	3.93
	Cronbach’s alfa	0.75	0.87

Note: Included in analysis n = 151. Coefficients below 0.10 are not shown. Factors are correlated with a coefficient of 0.48

##### Convergent validity -Self efficacy scale

The mean for the overall self-efficacy scale (including all items, range 0–80) was 47.8 (95 % BCa CI 44.4–51.3) for those with positive experience of talking to older patients about abuse and 40.4 (95 % BCa CI 37.6–43.1) for those with negative or no experience. The mean for the self-efficacy scale for asking questions (items 1–3, range 0–30) was 16.0 (95 % BCa CI 14.6–17.6) for those with positive experience and 12.4 (95 % BCa CI 11.3–13.5) for those with negative or no experience. Finally, the mean for the self-efficacy scale for managing response (items 4–8, range 0–50) was 31.8 (95 % BCa CI 29.3–34.3) for those with positive experience, and 28.0 (95 % BCa CI 25.9–30.1) for those with negative or no experience. All differences were significant (overall scale *p* < 0.01, self-efficacy in asking *p* < 0.01 and self-efficacy in managing *p* = 0.03).

##### Convergent validity - Cause for concern

The convergent validity of the items under Cause for concern is presented in Table 4. Reporting positive experience of talking to older patients about abuse was associated with less concern about negative reactions from patients and less concern for a negative effect on the patient-care provider relationship. No association was found between reporting positive experiences and the other items under cause for concern (Table 4).

**Table 4:** Convergent validity of cause for concern

	Experience of talking about abuse				
	**No, do not remember, negative**		**Yes, positive**		**P**
**Cause for concern**	**n**	**%**	**n**	**%**	
**Negative reaction from patient**					0.04
Not at all concerned	13	14.3	12	24.0	
Little concerned	53	58.2	31	62.0	
Rather or very concerned	25	27.5	7	14.0	
**Negative impact on relationship**					0.02
Not at all concerned	26	28.6	20	40.0	
Little concerned	40	44.0	25	50.0	
Rather or very concerned	25	27.5	5	10.0	
**Patient’s situation gets worse**					0.24
Not at all concerned	19	20.9	14	28.0	
Little concerned	45	49.5	25	50.0	
Rather or very concerned	27	29.7	11	22.0	
**Dangerous or threatening situations for me**					0.22
Not at all concerned	49	53.8	33	66.0	
Little concerned	30	33.0	12	24.0	
Rather or very concerned	12	13.2	5	10.0	
**Not be able to offer a good follow up**					0.39
Not at all concerned	7	7.7	8	16.0	
Little concerned	37	40.7	18	36.0	
Rather concerned	31	34.1	16	32.0	
Very concerned	16	17.6	8	16.0	

Note: *P* Significance level of chi-square test for linear trend. No experience or negative *n* = 93, positive experiences *n* = 50, missing cases *n *= 11. Cause for concern, missing cases *n* = 1–3

Self-efficacy in managing abuse was negatively correlated with concern about not being able to offer the patient a good follow up (Spearman correlation − 0.30, p < 0.01) i.e., respondents with strong self-efficacy tended to report less concern. Also as expected, self-efficacy for asking questions was negatively correlated with concern for negative reactions (Spearman correlation − 0.33 *p* < 0.01) and a negative effect on the relationship (Spearman correlation − 0.18, *p *= 0.03).

##### Convergent validity - Organizational and structural barriers

The majority (73.7 %) of respondents with experience of talking to patients about abuse were aware of the readiness of their workplace to manage cases, compared to 42.1 % of those without experience (*p* < 0.01). Likewise, 67.1 % of those with experience and only 51.3 % of those without experience knew about the preparedness of society (*p* = 0.048) to support older adults subjected to abuse. A strong overall self-efficacy was correlated with high confidence in ability to document elder abuse cases (Spearman correlation coefficient 0.16, *p* = 0.045) and high perceived knowledge about legislation on elder abuse (Spearman correlation coefficient 0.32, p < 0.01). Also, those who knew which colleague they would turn to if they needed help in managing elder abuse cases had a significantly (*p* = 0.03) higher mean on the overall self-efficacy scale (43.8, 95 % BCa CI 41.4–46.2) compared to those who did not know whom to turn to (38.6, 95 % BCa CI 34.3–43.0). Reporting to have more time to ask older patients about abuse was positively correlated with self-efficacy in asking questions (Spearman correlation coefficient 0.17, *p* = 0.04).

## Phase II: Revision of REAGERA-P and test of responsiveness

Phase I indicated that REAGERA-P had good validity, except for the case vignette that showed non-satisfying results. Phase II was therefore performed to test a revised version of the case vignette. Also, we wanted to test the responsiveness of the self-efficacy scale and of the items under cause for concern, i.e., changes in responses before and after an educational intervention intended both to increase self-efficacy and to lessen cause for concern. If the items would show good responsiveness, we intended to use them as outcome measures in future educational interventions on elder abuse.

The version of REAGERA-P used in phase II included the new case vignette, the self-efficacy scale, cause for concern, and some new questions, e.g., more elaborate questions about own previous experience and questions to evaluate the educational intervention. The new questions will not be further elaborated on here. Because we did not want to increase the answering time and because we did not expect organizational barriers to change before and after an educational intervention, we decided to exclude the items about organizational barriers in phase II. The final version of REAGERA-P is presented as a supplementary file.

### Method phase II

In the case vignettes used in phase I only one symptom was provided for each case and a large proportion of respondents choose to answer that they would need more information before they would ask questions about abuse. The new case vignette was therefore constructed in a different way where gradually more indicators of possible abuse were presented in an effort to capture a level of information that triggered the respondent to take further action. After each piece of new information was given, the respondent was asked if they, given all the information they had, would ask the patient questions about abuse. Response options were (a) not at all likely, (b) not very likely, (c) fairly likely, and (d) very likely. When the respondent answered “very likely,” they were not asked any more questions about the case. Respondents more likely to ask questions early in the case vignette were considered more aware of elder abuse. By constructing the case vignette in this way, we also intended that it can be used repeatedly and possibly be a way to measure changes in awareness over time. The symptoms added to each step are described in Table 5 and the entire case vignette is included in the supplementary file.

**Table 5:** Likelihood of asking about abuse based on the information in the case vignette

		Not at all likely		Not very likely		Fairly likely		Very likely	
**Step**	**Added information**	**N**	**%**	**N**		**N**	**%**	**N**	**%**
1	Frequent attender with well investigated abdominal pain	11	35.5	20	64.5	-	-	-	-
2	History of unexplained chest pain and backpain	6	19.4	19	61.3	6	19.4	-	-
3	Previous overdose of antidepressant under unclear circumstances	4	12.9	14	45.2	12	38.7	1	3.2
4	Financially dependent son has moved in and thinks that home care is unnecessary	1	3.2	8	25.8	14	45.2	7	22.6
5	Unexplained bruises on upper arms	-	-	3	9.7	7	22.6	13	41.9

Note: When respondent answered “very likely” they were not asked any more questions about the case vignette, consequently, one respondent was excluded after step one, one more was excluded after step three, and seven more were excluded after step four. Percentages are calculated for the original number of respondents (n = 31)

#### Cognitive interviews

A web version was used for the data collection in the second phase, in contrast to the first phase in which a questionnaire on paper was used. Five new cognitive interviews were performed to examine cognitive process and to ensure comprehension of questions and usability of the web version The same technique as previously described for cognitive interviews were used. Three informants responded to the REAGERA-P on a cell phone and two used a computer. All informants found the legibility of the questions on the different devices to be satisfying. The new case vignette was well understood, and no changes had to be made. The informants, however, found the questions about concern that a patient situation would worsen or concern that a caregiver would end up in a dangerous situation as less relevant than the other items under cause for concern. These two items were excluded as only a few respondents expressed strong concern about the items in the first data collection, and we wanted to restrict the number of questions. Some of the informants also found that it was more logical to ask questions about cause for concern directly after the case vignette, before the self-efficacy scale, and we therefore changed the order of those parts of the questionnaire. The new cognitive interviews also resulted in a few minor linguistic improvements to items, but no major changes.

The exact wording of questions in the final version of REAGERA-P is presented in a supplementary file. It was translated into English by a professional translator and then translated back to Swedish by a second professional translator. Final adjustments of the wording of the English version were made in agreement among the translators and the researchers.

#### Test of responsiveness

Eligible were medical interns (*n* = 39) who participated in an educational intervention on elder abuse in the fall of 2020. Participants used their cell phones to respond to the questionnaire twice during the day, before and after the intervention. It took on average about 10–15 min to answer the questionnaire both before and after the intervention. One person declined participation, three persons did not participate in the data collection before the intervention, while four persons did not participate afterwards. In total, 31 participants responded on both data collection occasions and were included in the analyses (response rate 79.5 %). The background characteristics of the samples in both phase I and II are presented in Table 2.

Internal consistency for the self-efficacy scale was calculated for the new sample. Thereafter, a paired sample T-test with bootstrapping and 95 % BCa CI for 1000 samples was used to compare means before and after the intervention. We used Wilcoxon’s signed-rank test for two related samples to evaluate changes in the items on concern about elder abuse.

### Results – Phase II

#### Performance of case vignette

Table 5 shows that the initial testing of the new vignette indicated that it performed as intended. As more information was obtained, a larger percentage of respondents answered that they would be more likely to ask questions.

#### Responsiveness of Self-efficacy scale and cause for concern

The Cronbach’s alfa for the total self-efficacy scale was 0.73 before the intervention and 0.81 afterwards. The Cronbach’s alfa for the scale for managing the response was satisfying with 0.74 before the intervention and 0.76 afterwards, but the Cronbach’s alfa for the scale for asking questions about abuse was less satisfying with 0.51 before the intervention and 0.49 afterwards. The self-efficacy scales showed good responsiveness, i.e., the mean on the scale was significantly higher after the intervention than before (Table 6).

**Table 6:** Responsiveness of the self-efficacy scale and items about cause for concern

	Before intervention		After intervention		p			
**Self-efficacy**	**Mean**	**95 % BCa CI**	**Mean**	**95 % BCa CI**				
Overall scale (range 0–80)	37.3	34.2–40.2	54.0	51.7–56.2	< 0.01			
Asking questions (range 0–30)	15.0	13.6–16.2	21.5	20.6–22.3	< 0.01			
Managing the response (range 0–50)	22.4	20.0-24.7	32.5	30.9–34.2	< 0.01			
**Cause for concern**	**n**	**%**	**n**	**%**	**p**	**Negative ranks (n)**	**Positive ranks (n)**	**Ties**
Negative reaction from patient					1.00	5	5	21
Not at all worried	15	48.4	10	32.3				
Little worried	11	35.5	21	67.7				
Rather worried	5	16.1	1	2.9				
Very worried	0	-	0	-				
Negative effect on relationship					0.97	6	8	17
Not at all worried	15	48.4	10	32.3				
Little worried	12	38.7	20	64.5				
Rather worried	2	6.5	1	3.2				
Very worried	2	6.5	0	-				
Not be able to offer a good follow up					0.54	13	7	11
Not at all worried	6	19.4	1	3.2				
Little worried	8	25.8	17	54.8				
Rather worried	12	38.7	13	41.9				
Very worried	5	16.1	0	-				

Note: *BCa CI* Bias corrected accelerated confidence intervals (BCa CI) for 1000 samples. *P*-value for self-efficacy = T-test for dependent samples. *P*-value for cause for concern = Wilcoxon’s signed-rank test for two related samples

Changes in responses were not significant between before and after measurements for any of the three items on cause for concern (Table 6). The majority of respondents gave the same answer before and after the intervention for negative reactions (*n* = 21 of 31, 68 %) and negative relations (*n* = 17 of 31, 55 %). A greater difference was found in responses to the question about cause for concern under being able to give the patient a good follow up. Thirteen participants reported less concern and 7 reported more concern post intervention, while 11 reported the same as before the intervention (Table 6).

## Discussion

In this article, we described the development and testing of REAGERA-P, a new questionnaire that can be used to measure health care provider preparedness to identify and manage cases of elder abuse. The questionnaire showed good validity, and the test of the included self-efficacy scale also indicated good responsiveness.

The different parts of the REAGERA-P were evaluated in different ways. Content and face validity were ensured for all items by using a review committee, an expert review, and cognitive interviews. Factor analysis and measurements of internal consistence (Cronbach’s alpha) were calculated for the self-efficacy scale. The other parts, intended to be used as separate items rather than scales, were tested for convergent validity, i.e., tested for associations among the items of the questionnaire. It would have been preferable to test convergent validity against another well-validated measurement of the same construct. However, the major reason for developing REAGERA-P was the lack of previously validated questionnaires, and such comparisons were, therefore, not possible. The procedure we used has been recommended for evaluating self-efficacy scales as a way of testing if respondents differ in their response in predictable and theoretically sound ways. [39].

The different parts of the questionnaire were intended to be used separately or together, depending on the aim of the data collection. The self-efficacy scale and the questions about cause for concern were tested in both phase I and II, but there were several methodological differences between the two occasions of data collection: (a) a paper questionnaire in phase I and a web survey in phase II, (b) different case vignettes were used, (c) the order of the questions was revised in phase II, (d) the questions following the self-efficacy scale were different in the two phases, and (e) the study population in phase I consisted of mixed professions, while only early career physicians were included in phase II. Despite all these differences, the overall self-efficacy scale and the subscale for self-efficacy in managing cases showed good internal consistency in both phases, indicating that the scale is a valid measurement of self-efficacy also when used in different contexts. The Cronbach’s alpha for the subscale for self-efficacy in asking about abuse was not as satisfying in phase II. It is not possible to discern from our study the reason for the inferior internal consistency, but the small sample size in the second phase in combination with the few items of the scale likely played a role. The questions on cause for concern were also included in both phases and similar distributions of answers were found in the two datasets indicating that the methodological differences between the two phases previously mentioned were not a major concern. However, it is possible that the case vignette preceding the other questions affected the mindset of respondents. This is supported by anecdotal evidence of comments from respondents who said that they had not really thought of the symptoms presented as possible signs of abuse, but that they did so after reading the case vignette. The case vignette was intended to measure awareness but might in fact have had an intervention effect by increasing awareness among respondents. Hence, though we recommend that REAGERA-P should be used as an entity, we conclude that it is likely that the different parts of the questionnaire can be used independently in future data collections depending on the aim of the study. However, caution is necessary if the case vignette is excluded.

We expected that previous predominantly positive experience of talking to patients about elder abuse would be associated with less concern for not being able give the patient a good follow up, but it was not. The reason we found no association might be that the question about experience was interpreted as pertaining to verbal conversation with the patient and not to what happens after the encounter. Also, only a few respondents reported good preparedness to care for older adults subjected to abuse at their workplace and in society, and there were no clear guidelines at the clinic for how to manage victims of elder abuse at the time of the data collection. Therefore, concern about not being able to give the patient a good follow up might be grounded in not knowing how to best manage the patient because of a lack of experience, but concern could also be grounded in previous experience of managing cases without any guidelines. Consequently, the lack of association does not necessarily mean that the concern question is invalid. This interpretation was supported by the fact that the item on self-efficacy for managing cases was negatively correlated with concern about not being able to give the patient a good follow up.

The self-efficacy scale was found to have good responsiveness, but the questions about cause for concern did not. We expected the intervention to be associated with less concern, and the reasons why it was not remain unclear. The educational intervention might not have been sufficiently effective in reducing concern, but the limited reduction in concern might also reflect a combination of increased self-efficacy among the participants and a parallel increased awareness about the complexity of elder abuse. Participants may have realized shortcomings in the health care system’s response to cases of elder abuse that they had not previously been aware of. Hence, it is possible that for some participants who had little concern before the training course, learning about elder abuse opened their eyes to the difficulties of managing cases and the lack of evidence-based approaches to manage cases, which may have led to more cause for concern. This possible explanation is supported by the findings presented in Table 6, which show that fewer participants reported extreme values (not at all worried and very worried) after the course, while more participants reported that they were a “little worried.” In other words, it is possible that the questions had good responsiveness but that the educational intervention did not influence participants in the expected way. The different items under cause for concern have been firmly supported in previous research [11, 15, 20]. We also found an association between these items and the positive experience of talking to older patients about abuse in phase I. Consequently, we recommend that the items should be used but should be further tested for responsiveness in other interventional studies.

The most important outcome measure for a questionnaire of this kind would be if the responses to the questions predict later behavior, e.g., does high self-efficacy or a low level of concern predict the likelihood of identifying and managing cases of elder abuse? In his instructions for constructing self-efficacy scales, Bandura underlines that self-efficacy is a judgment of capability and while self-efficacy is theorized to be a major determinant of intention, self-efficacy and intention are separate concepts [39]. Consequently, although we found good responsiveness for the self-efficacy scale, it is not clear if increased self-efficacy in asking about abuse equals higher identification rates. However, an association between strong self-efficacy and a higher screening rate has previously been reported in a study about intimate partner violence [36]. This question needs to be considered in future longitudinal studies using the REAGERA-P.

All questionnaires are to some extent culturally and context dependent. The support system for older adults, and legislation on elder abuse differ among countries. For example, legislated mandatory reporting of elder abuse and adult protective services is the norm in the many states in the US. In line with this, several previous questionnaires have focused on reporting practices [29, 30, 32]. As we do not have mandatory reporting legislation in Sweden, we decided to use a single, generic question in REAGERA-P about perceived competence regarding legislation and regulations on elder abuse. That question is presumably less dependent on culture and context, but this calls for investigation in future research. Previous questionnaires have also focused to a large extent on knowledge about elder abuse [32]. However, measuring knowledge is difficult in an area where so many aspects lack solid scientific ground. For example, some previous studies have measured knowledge by asking respondent to evaluate if the following statements are “true,” “false” or “don’t know”: “Very few older adults are abused”, “Experienced persons in my profession can accurately diagnose cases of elder abuse,” and “Most older people are able to get help if they need it” [30, 32]. As no validity measures for these questions have been reported, it is unclear how respondents interpreted the questions. What do “very few,” “experienced persons,” “accurately diagnose,” or “get help” actually mean? We did not include questions to measure respondents’ knowledge about facts, instead we chose to ask about their knowledge about the preparedness of their workplace and society to care for victims of elder abuse. More than 40 % of respondents did not know about the preparedness of society or their workplace to care for victims of elder abuse. This indicates that many health care professionals do not know about the available support systems, which is a finding in line with previous research that has reported low awareness of elder abuse among health care professionals [12–14, 31]. However, it should also be underlined that there is a lack of support systems available for older adults subjected to abuse in Sweden, which is likely also reflected in these answers.

### Limitations

A potential limitation is that the data collection included health care providers employed at only one hospital. However, the sample included respondents with different professions, sex, age and working experience which may be more methodologically relevant for testing a questionnaire. For phase II we had planned a larger data collection, but this could not be performed due to restrictions for public gatherings during the Covid-19 pandemic. However, despite the small sample size, differences in self-efficacy before and after the educational intervention were significant, indicating a good responsiveness for the scales.

The first and last authors were employed at the clinic where phase I of the study took place, which might have affected the respondents’ willingness to participate or their responses. To minimize these risks, the data collection was done anonymously, and we made efforts to not use questions which could reveal identity e.g., we asked about age categories instead of specific age.

The study was performed in Sweden and generalizability of the results to other countries is uncertain. Ideally all instruments should be validated in the language and cultural context for which they are intended. For example, support systems and legislations surrounding elder abuse are different in different countries which might affect both the relevance of specific questions and how they are interpreted by respondents. In Sweden, societal attention to elder abuse has gradually increased but from a very low level, a reality that was also reflected in our findings. Hence, although we believe that REAGERA-P is usable in other countries, this need to be evaluated in future studies.

## Conclusions

This article described the development and validity testing of REAGERA-P, a new questionnaire to evaluate health care professional preparedness to identify and manage elder abuse. The questionnaire showed good content and face validity as well as good construct validity and responsiveness for relevant scales and parts in this initial testing. Further research is needed to study if the REAGERA-P can be used to predict later behaviors, e.g., if it increased the respondent’s ability to identify and manage cases of elder abuse. More research is also needed to investigate how the health care response to victims of elder abuse can be improved. REAGERA-P can be a valuable questionnaire to assess provider preparedness in different kinds of research, such as an outcome measure in educational intervention studies.

## Supplementary Information

**Additional file 1.**

## Data Availability

The datasets used and analyzed during the current study are available from the corresponding author on reasonable request.

## Abbreviations

REAGERA-P: Responding to Elder Abuse in GERiAtric care – Provider questionnaire
BCa CI: Bias corrected accelerated confidence intervals
