# Supplementary material for: Development and validation of REAGERA-P, a new questionnaire to evaluate health care provider preparedness to identify and manage elder abuse
Source: BMC Health Serv Res. 2021 May 19;21:473. doi: 10.1186/s12913-021-06469-2 (PMC8131191; doi:10.1186/s12913-021-06469-2)
Supplement: Supplementary file 1 — Additional file 1. [file 12913_2021_6469_MOESM1_ESM.docx]

## **REAGERA-P**

## Background characteristics

1. **Are you**

- Female
- Male
- Other

1. **How old are you?**

- Up to 34 years old
- 35-49 years old
- 50 years old or older

1. **What is your current profession?**

- Assistant nurse
- Nurse
- Physician
- Other

1. **How long have you worked in your current profession?**

- Less than one year
- One to five years
- Five or more years

1. **How long have you worked at your current workplace?**

- Less than one year
- One to five years
- Five or more years

1. **In your education, did you receive training on violence in close relationships (regardless of age) or elder abuse?** (Multiple answers possible)

- Yes, elder abuse
- Yes, violence in close relationships
- No
- Do not remember

1. **Did you at any other time receive training on violence in close relationships (regardless of age) or elder abuse?** (Multiple answers possible)

- Yes, elder abuse
- Yes, violence in close relationships
- No
- Do not remember

**Abuse of an older person includes:**

- **physical, emotional and sexual violence or abuse**
- **financially exploitation**
- **neglect**

**By older, we mean individuals over 65 years of age.**

**By “asking questions about abuse”, we mean that you directly ask if the patient has been treated badly or subjected to some kind of abuse. Accordingly, we do not mean general questions about circumstances at home or how they are doing.**

**This applies to the entire questionnaire.**

## PATIENT CASE

*You will now be asked to read a patient case. In your work, what would you think about asking this patient questions about abuse in different phases of your contact?*

Gunnel, aged 77, is admitted to the hospital due to a deterioration of her COPD. Her breathing rapidly improves, but Gunnel instead complains a lot about abdominal pain. She has sought care for this several times both at the health care centre and the emergency ward, but the pain does not improve. She previously underwent a thorough investigation, including gastroscopy, without any explanation for the symptoms being found.

[Alternative text health care centre: Gunnel, aged 77, has recently registered with the health care centre and you meet her for the first time for an annual exam of her COPD. It seems to be well-managed, but Gunnel instead complains a lot about abdominal pain.]

**11. How likely is it, based solely on this information, that you ask Gunnel questions about abuse?**

- Not at all likely
- Not particularly likely
- Somewhat likely
- Very likely

In the conversation, it comes forth that Gunnel in recent years has sought care on multiple occasions with different symptoms, but no good explanation has been found for her symptoms. Among other things, she was treated for chest pain that was not deemed to be cardiac related, and she has had very troublesome back pain for an unclear reason.

1. **Based on the information you now have access to, how likely is it that you ask Gunnel questions about abuse?**

- Not at all likely
- Not particularly likely
- Somewhat likely
- Very likely

Before the next time you see Gunnel, you see in the medical records that she has been depressed periodically. Last year, she received in-patient care over 24 hours because she had taken too many of her antidepressive pills. In the medical record, it says that the overdose was probably happened by mistake, but that the circumstances were a little unclear. After that care episode, Gunnel received a dose-dispensing service for her medications, so that it would not happen again.

1. **Based on the information you now have access to, how likely is it that you ask Gunnel questions about abuse?**

- Not at all likely
- Not particularly likely
- Somewhat likely
- Very likely

Gunnel says that she is single and lives in a villa. She has handled it well so far, but she says that she would need home-help services now to be able to manage everything. Gunnel has a son who lives in the same city and he has financial problems and therefore lives with Gunnel now and then. When you see Gunnel, you ask if she likes having her son living with her sometimes. Gunnel answers vaguely and evasively. A few days later, a needs assessment is done and the son then says that he thinks it is unnecessary to spend money on the home-help services, and Gunnel agrees. Afterwards, you meet Gunnel alone again.

1. **Based on the information you now have access to, how likely is it that you ask Gunnel questions about abuse?**

- Not at all likely
- Not particularly likely
- Somewhat likely
- Very likely

You now also examine Gunnel again and note something that you had not seen before. She has older bruises on both upper arms. When you ask what happened, Gunnel tries to joke the question away and says that she does not know, but that she might have “happened to bump into something”.

1. **Based on the information you now have access to, how likely is it that you ask Gunnel questions about abuse?**

- Not at all likely
- Not particularly likely
- Somewhat likely
- Very likely

## Cause for concern

1. **How concerned are you about the following things when it comes to asking older patients questions about abuse?**
2. **That the patient reacts negatively if I ask questions**

- Not at all concerned
- A little concerned
- Somewhat concerned
- Very concerned

1. **That the patient-care provider relationship will be negatively impacted if I ask questions**

- Not at all concerned
- A little concerned
- Somewhat concerned
- Very concerned

1. **That I will not be able to offer the patient a good follow-up**

- Not at all concerned
- A little concerned
- Somewhat concerned
- Very concerned

## Self-efficacy

1. **At present, how would you manage to do the following things in your work?**

| Would magage it very poorly |  | Would manage it very well |
| --- | --- | --- |
| 0 1 2 | 3 4 5 | 6 7 8 9 10 |

- 1. Asking questions about abuse to an older patient who has clear indications of now being, or having previously been, subjected to abuse
  2. Asking questions about abuse to an older patient who has no clear indications of now being or having previously been, subjected to abuse.
  3. Ensuring you are able to ask questions about abuse in private to an older patient who has a relative who insists on being present during all contact
  4. In conversation, providing support to an older patient who tells about abuse
  5. Helping an older patient subjected to abuse on to the right body in healthcare, or to the right support function in society
  6. Helping an older patient subjected to abuse to make a report to the police or social services
  7. Helping and supporting an older patient subjected to abuse, who does not currently want to change his or her situation

h. Handling the meeting with an older patient

who says no to questions about abuse, but

where you still have strong suspicions that

the patient is subjected to abuse.

## Questions about own previous experiences

1. **How many times in the past six months has an older patient spontaneously told you about experiencing abuse, without you asking questions about it?**

- None
- Once
- 2-4 times
- 5 times or more
- Do not remember

1. **How many times have you asked older patients questions about abuse in the past six months?**

- None
- Once
- 2-4 times
- 5 times or more
- Do not remember

1. **How many times did the questions lead to an older patient telling about abuse that he or she experienced?**

- None
- Once
- 2-4 times
- 5 times or more

## Questions about organizational conditions and potential improvements

1. **In your current work situation, how often do you have time to bring up the issue of abuse of older people with your patients if you would like to?**
   - - Never
     - Rarely
     - Often
     - Always
2. **If you would like help to handle the situation when an older patient tells you about abuse, do you know who at your workplace you could turn to?**
   - - Yes
     - No
3. **How do you think the preparedness at your workplace is for taking care of older patients subjected to abuse?**
   - - Very good
     - Fairly good
     - Somewhat inadequate
     - Very inadequate
     - Don’t know what preparedness there is
4. **How do you think the preparedness in society is for taking care of older patients subjected to abuse?**
   - - Very good
     - Fairly good
     - Somewhat inadequate
     - Very inadequate
     - Don’t know what preparedness there is
5. **Do you know what you should do to document what patients tell you about abuse in a correct and secure way in the medical record?**

- Absolutely
- To a large extent
- To some extent
- Not really

1. **Do you think you have enough legal knowledge, for example about when and to whom one can/must report if an older patient is mistreated and what secrecy rules apply?**

- Absolutely
- To a large extent
- To some extent
- Not really

1. **How much responsibility do you think the following professional categories have at your workplace for asking older patients questions about abuse?**

|  |  | None | Fairly little | Quite a lot | A lot |
| --- | --- | --- | --- | --- | --- |
|  | Nurse |  |  |  |  |
|  | Assistant nurse |  |  |  |  |
|  | Occupational therapist, physiotherapist |  |  |  |  |
|  | Physician |  |  |  |  |

1. **How much would the following measures make it easier for you in terms of handling the encounter with older persons subjected to abuse?**

|  |  | None | Fairly little | Quite a lot | A lot |
| --- | --- | --- | --- | --- | --- |
| a. | Appointment of a resource person with special responsibility for helping with managing such cases |  |  |  |  |
| c. | Training in how to ask questions about abuse |  |  |  |  |
| d. | Training in what support services are available in society |  |  |  |  |
| e. | Written guidelines for the staff that include contact information to the support services available |  |  |  |  |
| f. | A written document directed at patients containing contact information to the support services available in society |  |  |  |  |
